# Supplementary material for: Changes in the Human Gut Microbiome during Dietary Supplementation with Modified Rice Bran Arabinoxylan Compound
Source: Molecules. 2023 Jul 14;28(14):5400. doi: 10.3390/molecules28145400 (PMC10385627; doi:10.3390/molecules28145400)
Supplement: Supplementary file 1 [file molecules-28-05400-s001.zip › molecules-2450332-supplementary.pdf]

Supplementary S1

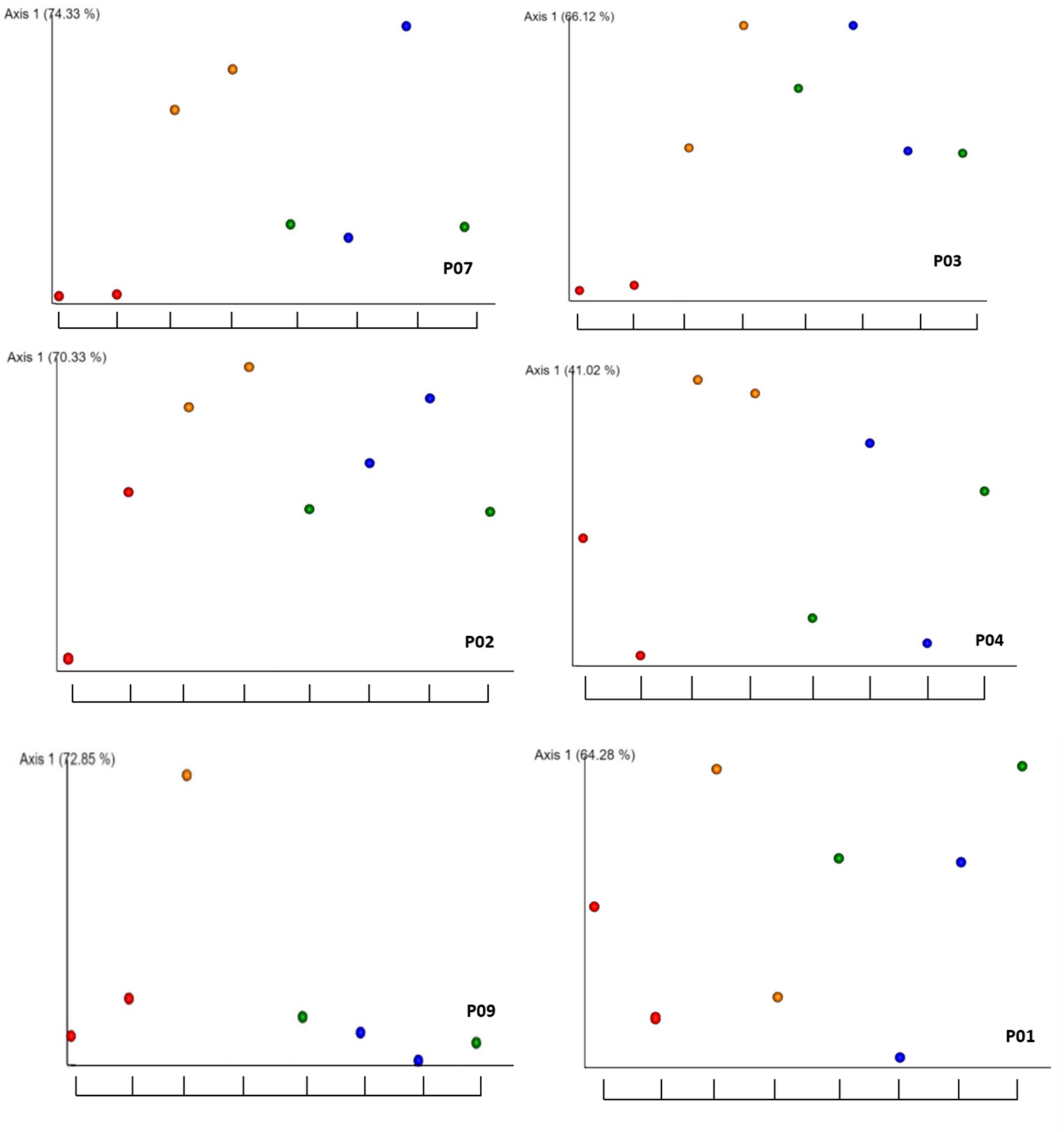

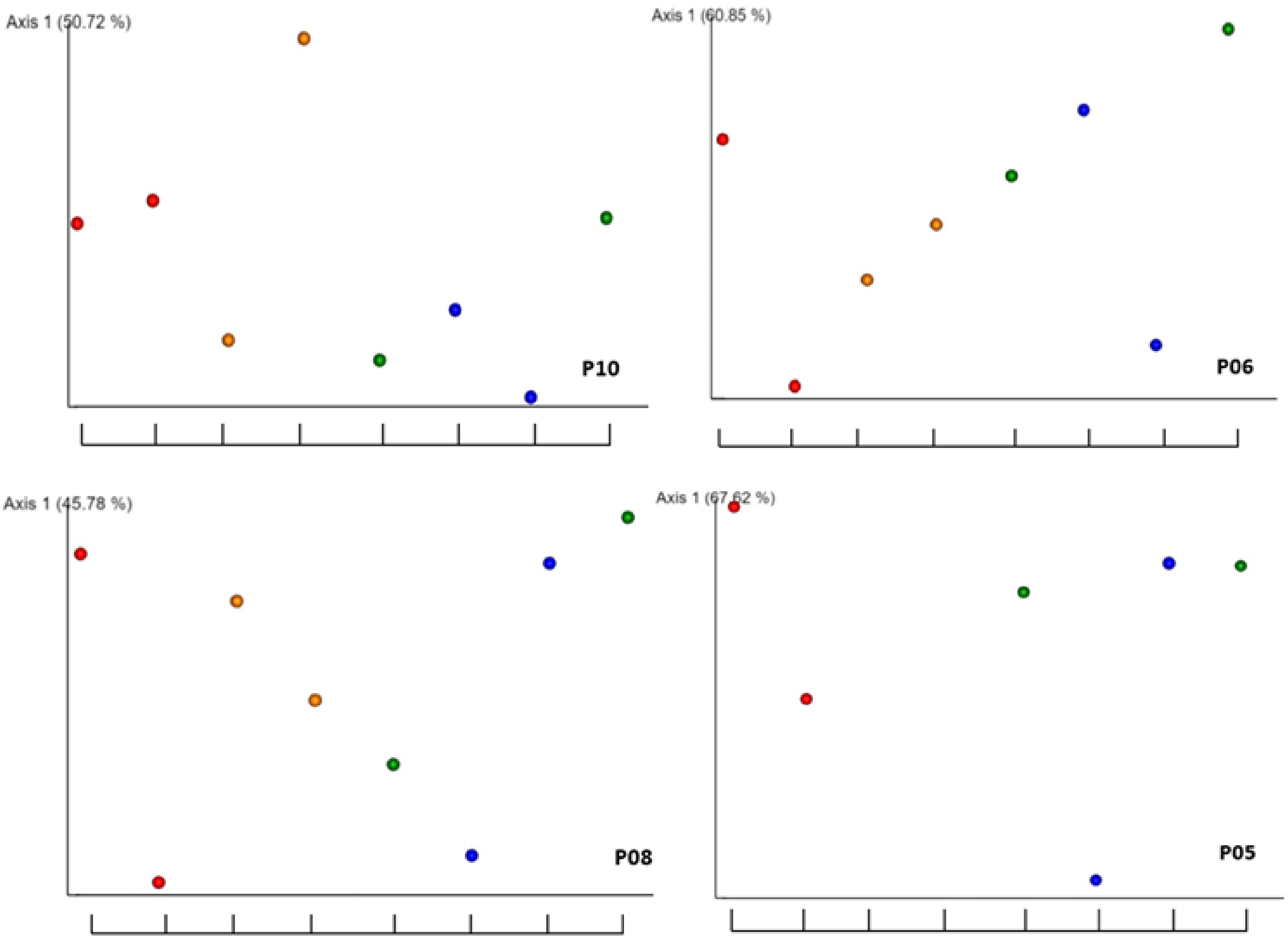

Figure S1: EMPeror plots of weighted unifrac beta diversity distance matrix of each participant. Axis 1 indicates the percentage of variation of the total variance between samples (alteration of microbial composition) and X-axis indicates days since experimental phase began, depicting time points 1-8. Distance on Y-axis indicates similarity of samples. Each interventional phase has a total of 2 time points except washout and post-intervention which have 1 time point each. Multiple samples on the same time point indicate a technical replicate and missing samples are not depicted.

Legend:

Baseline

Low dose

Washout & post-intervention

High dose
